# MOG antibody-associated encephalitis secondary to Covid-19: case report

**DOI:** 10.1186/s12883-021-02449-5

**Published:** 2021-10-27

**Authors:** Ervin Durovic, Corinna Bien, Christian G. Bien, Stefan Isenmann

**Affiliations:** 1 Department of Neurology, Klinik für Neurologie und klinische Neurophysiologie, St. Josef Krankenhaus Moers, Asbergerstr. 4, 47441 Moers, Germany; 2 Present Address: Laboratory Krone, Bad Salzuflen, Germany; 3 Department of Epileptology (Krankenhaus Mara), Bielefeld University, Medical School, Campus Bielefeld-Bethel, Bielefeld, Germany

**Keywords:** COVID-19, Encephalitis, MOG antibody, Methylprednisolone, Neuropsychology, SARS-CoV-2

## Abstract

**Background:**

While Covid-19 predominantly affects the respiratory system, neurological manifestations including encephalitis occur in some patients, possibly affecting the course and outcome of the disease. Here, we describe a unique case of a young man with Covid-19 and transient MOG-positive encephalitis, with a benign course.

**Case presentation:**

A 22-year-old male, with PCR confirmed Covid-19 infection was admitted because of persistent headache. The clinical examination was normal. Neuropsychological testing revealed distinct executive deficits. Brain MRI and cerebrospinal fluid (CSF) analysis were suggestive for encephalitis. Further laboratory examination revealed a serum MOG antibody titre. The headache improved with analgetic treatment and i.v. methylprednisolone. Consequently, the MOG antibody titer decreased and MRI lesions were resolving. The patient made a full recovery, with no signs of deterioration over the following months.

**Conclusions:**

Covid-19 manifestations in the CNS include encephalitis with variable course and prognosis. This case highlights a possible association between inflammation due to COVID-19 and transient secondary autoimmunity with transient MOG antibodies and atypical clinical presentation.

## Background

SARS-CoV-2 was first identified in December 2019 in Wuhan, China [1]. Soon, it became clear that severely affected patients may also develop neurological symptoms and complications [2], including meningoencephalitis [3, 4]. Although typically causing fever, respiratory symptoms and myalgia, neurological syndromes in association with SARS-CoV-2 that may affect the course of the disease and outcome include inflammatory diseases such as Guillain-Barré syndrome (GBS), Miller-Fisher syndrome (MFS), (meningo-) encephalitis, and myelitis, as well as stroke, seizures, and myopathy [4, 5].

## Case presentation

A previously healthy and asymptomatic 22-year-old male police officer was confirmed SARS-CoV-2 positive by PCR testing while in contact quarantine at home (= day 0). Ten days later, he was admitted to our hospital because of severe headache (verbal rating scale, VRS 8/10) that had started a week previously, few days after his positive test. He reported fever, neck stiffness, general weakness and a loss of smell and taste. He had been started on ASS, low-dose heparin, dexamethasone and aciclovir.

**Fig. 1:**
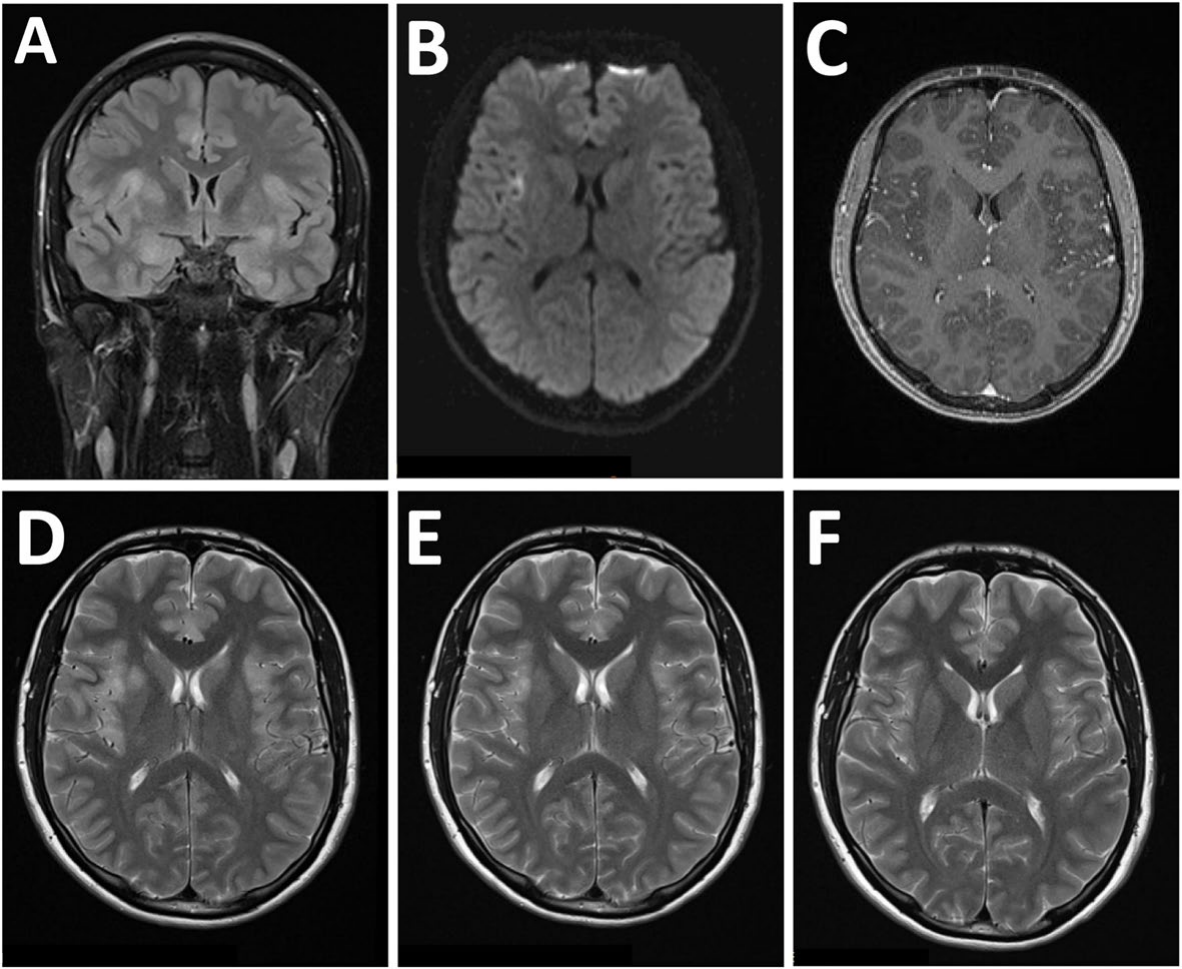
Serial MRI examinations. **A**-**D**, at initial examination (d 11 after PCR diagnosis), FLAIR (**A**) showed increased signal intensity bilaterally, in the cortex and basal ganglia. **B** DWI showed only a small area of restricted diffusion in the right insular cortex. **C** There was no signal enhancement after i.v. gadolinium. **D**-**F** T2 images showing signal hyperintensities before (d 11) (**D**) and improvement after 5 days of i.v. methylprednisolone (d17; **E**). **F** Full resolution (d 35)

**Fig. 2:**
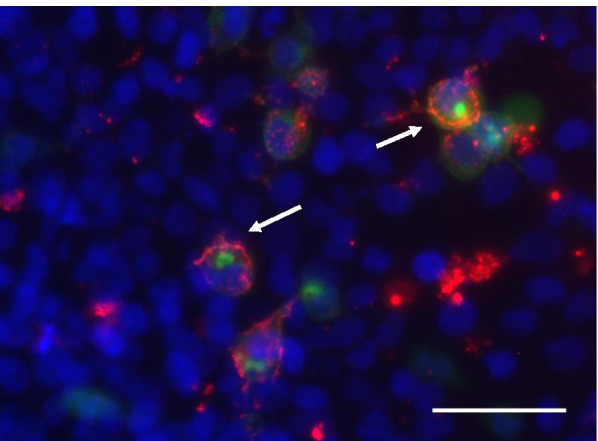
Live cell assay for the determination of antibodies against MOG. MOG-transfected human embryonic kidney cells (HEK, marked by the co-transfected intracellular green fluorescent protein [6] are bound by the MOG antibodies visualized by a red fluorochrome. Nuclear counterstaining in blue. Bar: 20 μm

On physical examination at admission, he was fully oriented and had some neck rigidity. Further neurological examination was normal. Blood laboratory tests were unremarkable. Brain MRI (day 11) showed multiple disseminated pathological T2 and FLAIR hyperintensities, predominantly cortically, without any contrast enhancement (Fig. 1 A-D). Comprehensive neuropsychological assessment on day 16 showed no memory impairment, but mild impairment in executive functions (Table 1). CSF analysis revealed a pleocytosis with 31 cells per μl (normal range, nr, < 5). Total CSF protein was 39.9 mg/dL (nr, 15–45), CSF glucose 64 mg/dL (nr, 40–70), and lactate 11.8 mg/l (nr, 10–22). There was no intrathecal IgG synthesis An extensive search for infectious, paraneoplastic, and autoimmune causes of encephalitis was performed. SARS-CoV-2 and HSV 1, 2 PCR in the CSF were negative, as was serology for Lyme borreliosis and HIV. Serum and CSF studies revealed a serum myelin oligodendrocyte glycoprotein (MOG) antibody titre of 1:640 (live-cell assay, see Fig. 2), and a low metabotropic glutamate receptor 1 (mGluR1) antibody titre (fixed cell assay, 1:40; see also Table 2). Dexamethasone and aciclovir were discontinued. One thousand milligrams methylprednisolone i.v. per day was given for 5 days. The patient’s general condition and headache improved gradually after completing the treatment, corresponding to marked improvement in the brain MRI on day 17 (Fig. 1E). He was discharged home and gradually resumed work. At a follow-up visit (day 35), the patient felt well. There was no headache, no focal neurological deficits, and no meningism. Extensive follow-up neuropsychological assessment was entirely normal, with above average results in all dimensions (Table 1). Follow-up MRI showed a resolution of signal alterations (Fig. 1 F). The serum MOG antibody titre was reduced to 1:320, while further laboratory tests including mGluR1 antibody were normal. Upon telephone consultation after 2 months (day 63), he reported he felt well and was back to work in shifts, with no residual symptoms, no restrictions, and no medication.

**Table 1:** Neuropsychological tests

Day	Test (PR)	Result
d 16	TAP (66), WCST (84), DAT (86), VWM (63), 5-PT (68), CogFlex (97), RI (28),	all normal, no impairment
	ToL (24)	possible mild impairment
d 35	WAIS-IV (53), VLMT (45–78), RWT (88), ROCFT (96), TMT (60), WCST (90), FSMC (31), TAP (27,33)	all normal, no impairment

*PR* Percentage rank [normal = no impairment: ≥ 25)

*TAP* Test of Attentional Performance, *WCST* Wisconsin-Card-Sorting-Test, *DAT* Divided Attention Test, *VWM* Verbal Working Memory, *5-PT* 5-Point-Test, *CogFlex* Cognitive flexibility, *RI* Response Inhibition, *ToL* Tower of London (Planning ability), *WAIS-IV* Wechsler Adult Intelligence Scale, *VLMT* Verbal Learning and Memory Test, *RWT* Regensburg Word fluency Test, *ROCFT* Rey-Osterrieth Complex Figure Test, *TMT* Trail Making Test, *WCST* Wisconsin-Card-Sorting-Test, *FSMC* Fatigue Scale Motor and Cognition, *TAP* Test of Attentional Performance

**Table 2:** Serological examination and findings

Day	Serum	CSF	Immunoglobulin G ratio (serum/CSF)
d 11	anti-MOG, 1:640	anti-MOG, negative^a^	440
	anti-mGluR1, 1:40	anti-mGluR1, negative^a^	
d 35	anti-MOG, 1:320	n.d.	n.a.
	anti-mGluR1, 1:40	n.d.	

^a^Not demonstrable in undiluted CSF; n.d. = not done; n.a. = not applicable

*Antibodies tested in serum and CSF*: GAD65, NMDAR, GABAAR, GABABR, IgLON5, AMPAR1/2, DPPX, LGI1, CASPR2, glycin-receptor, MOG, mGluR5, mGluR1, amphiphysin, CV2/CRMP5, Ma2/Ta (PNMA2), Ri, Yo, Hu, recoverin, Sox1, titin, Zic4, DNER/Tr (for Methods, see [7])

## Discussion

We report a young male with PCR confirmed oligosymptomatic, clinically mild Covid-19 disease, and meningoencephalitis confirmed by brain MRI and CSF analysis. While several recent works reported on autoantibody findings in Covid-19 patients, this is, to our knowledge, the first report of a mildly affected adult with CSF and MRI confirmed meningoencephalitis and serum anti-MOG antibodies that decreased over time. The serum MOG antibody titre is above the threshold of 1:160 that has been suggested by experts in the field [8]. The lack of detectability in CSF is not surprising: The MOG antibody serum titre was 1:640, close to the immunoglobulin G ratio (CSF:serum) of 1:440. This means that only very few MOG antibodies crossed the blood-CSF barrier. It is known that MOG antibodies are usually not intrathecally synthesized [8]. Taken together, these pieces of information support the credibility to the MOG antibody titre results. According to an influential publication, a MOG antibody serum titre of 1:640 is “low positive” (1:160–1:640). For mGluR1 antibodies, no formal cut-off has been suggested. Nevertheless, by experience, the 1:40 serum titre appears too low to be relevant. Comparing this patient with the largest existing mGluR1 antibody series shows that he neither had the typical cerebellar ataxia nor the typical standard CSF abnormalities nor CSF antibodies against mGluR1 [9].

SARS-CoV-2 has only scarcely been found in the CSF, suggesting that Covid-associated neuropathology is caused by mechanisms different from direct viral toxicity [10]. Autoreactive antibodies targeting the CNS may mediate CNS and PNS damage. Thus, studies have looked for autoantibodies in Covid-19 patients. Franke et al. observed CSF-IgG binding to brain structures in all of their 11 severely ill Covid-19 patients with neurological symptoms examined, yet none with a defined antigenic target [11]. Recently, Fleischer et al. described neurological symptoms in 60% of their 102 SARS-CoV-2 positive patients. Of those tested, 35% were reported to harbor serum autoantibodies against diverse neuronal and glial epitopes (no titers given), without specific clinical correlations [12]. Without giving further details, or neuroimaging data, they did not find a correlation between severity of disease and presence of anti-MOG antibodies. In this case report, the MOG antibodies were tested by a live-cell assay and had a titre ≥1:160, which is an internationally accepted threshold for positivity [8, 13]. The monophasic disease course with multifocal fluffy T2/FLAIR hyperintense brain lesions correlated well to a MOG antibody-associated disease [13]. Vraka and colleagues reported a severely ill 13 months infant in the UK with altered consciousness, seizures, and fever [14]. The girl had received MMR vaccination a month before and had a febrile illness ten days previously. She required intubation and intensive care treatment. SARS-CoV-2 and adenovirus PCRs were positive, as were anti-MOG antibodies; there was no statement as to the method and titre. Cerebral MRI showed bilateral widespread white matter lesions including the splenium of the corpus callosum. She did not make a full recovery [14]. Due to the various confounders, it remains unclear if and how in this case MOG antibodies may have been connected to the SARS-CoV-2 infection, the MRI lesions, and the pathology in this infant [14]. Peters and co-workers recently presented the case of a 23-years old man with cognitive decline, unilateral headaches, dysesthesias and fever. After a positive SARS-CoV-2 test, he had generalized seizures. The brain MRI showed left hemispheric hyperintensity, as well as leptomeningeal enhancement. Serum MOG IgG antibodies were positive at 1:100. i. v. steroid treatment resulted in a marked improvement of cognitive function and MRI findings [15].

A Medline search (“[covid *or* covid-19 *or* SARS *or* SARS-CoV-2] *and* encephalitis *and* MOG”) conducted on 27 August 2021 yielded only two results (cited as refs. [14, 15] and discussed above), both published in 2021, and one review citing one case of MOG-associated myelitis. In addition, there are few heterogeneous case reports of optic neuritis with positive MOG-antibodies associated with Sars-CoV2 infection [16].

A recent systematic review and meta-analysis including all literature published until 24 October 2020 [17] found no reports on Covid-19-associated MOG positive encephalitis. However, in this systematic review, the average incidence of encephalitis a complication of covid-19 was estimated to be 0.2%, with a high rate of co-morbidities, and a mortality rate of 13.4% in affected patients [17].

Even though the primary target of SARS-CoV-2 is the respiratory system, the virus has also been recognized as a neuroinfectious agent. Several cases of possible encephalitis and para- or post-viral immune mediated neurological syndromes have been described in COVID-19 patients [18, 19]. Support for autoimmune mechanisms in “Neuro-Covid” also comes from a recent post-mortem case series reporting neuropathological changes predominantly in the brainstem and cerebellum, compatible with autoimmune encephalitis [20]. Potential pathogenetic mechanisms include molecular mimicry between viral proteins and neuronal autoantigens and delayed stimulation of post-viral autoimmunity similar to NMDA receptor encephalitis following herpes simplex virus (HSV) encephalitis [13].

MOG is a glycoprotein located on the myelin surface. The concept of inflammatory CNS disease associated with antibodies against MOG has evolved to include a wide variety of syndromes. MOG antibodies are more prevalent in demyelinating disorders (e.g., optic neuritis, ADEM), but can also be associated with encephalitis without demyelination [13]. However, little is known about secondary autoimmune encephalitis associated with SARS-CoV-2 infection. In view of this, in our case MOG antibodies might be either an immunological epiphenomenon or reflect possible secondary autoimmunity as yet another neurological feature of this deadly virus. It will remain crucial to collect further data on neurological manifestations worldwide, and to better understand the pathology and devise rational, pathogenesis-oriented treatment.

## Conclusion

We describe a unique case of a young man with Covid-19 and transient MOG-positive encephalitis, with a benign course. Covid-19 manifestations in the CNS include encephalitis with variable course and prognosis. This case highlights a possible association between inflammation due to COVID-19 and transient secondary autoimmunity.

## Data Availability

The data generated or analyzed during this study are included in this article.

## Abbreviations

SARS-CoV-2: Severe acute respiratory syndrome coronavirus 2
MOG: Myelin oligodendrocyte glycoprotein
mGluR1: metabotropic glutamate receptor 1
CSF: Cerebrospinal fluid
VRS: Verbal rating scale
GBS: Guillain-Barré syndrome
MFS: Miller-Fisher syndrome
MMR: Measles, mumps, and rubella
ADEM: Acute disseminated encephalomyelitis
PCR: Polymerase chain reaction
MRI: Magnetic resonance imaging
FLAIR: Fluid-attenuated inversion recovery
NMDA: N-methyl-D-aspartate receptor
